# Thyroid cancer in children: A multicenter international study highlighting clinical features and surgical outcomes of primary and secondary tumors

**DOI:** 10.3389/fped.2022.914942

**Published:** 2022-07-22

**Authors:** Cristina Martucci, Alessandro Crocoli, Maria Debora De Pasquale, Claudio Spinelli, Silvia Strambi, Paolo Brazzarola, Eleonora Morelli, Jessica Cassiani, Juliana Mancera, Juan Pablo Luengas, Pablo Lobos, Daniel Liberto, Estefanìa Astori, Sabine Sarnacki, Vincent Couloigner, François Simon, Cassandre Lambert, Simone de Campos Vieira Abib, Onivaldo Cervantes, Eliana Caran, Diana Delgado Lindman, Matthew O. Jones, Rajeev Shukla, Paul D. Losty, Alessandro Inserra

**Affiliations:** ^1^Department of Pediatric Surgery, Bambino Gesù Children's Hospital, IRCCS, Rome, Italy; ^2^Department of Pediatric Hematology/Oncology Cell and Gene Therapy, Bambino Gesù Children's Hospital, IRCCS, Rome, Italy; ^3^Department of Pediatric Surgery, University of Pisa, Pisa, Italy; ^4^Department of Surgery and Oncology, University and Hospital Trust of Verona, Verona, Italy; ^5^Department of Hepatobiliary Surgery, University of Verona, Verona, Italy; ^6^Department of Pediatric Surgery, Universidad Militar Nueva Granada, Bogotá, Colombia; ^7^Department of Pediatric Oncology, National Cancer Institute, Liverpool, Colombia; ^8^Department of Pediatric Surgery, Hospital Italiano de Buenos Aires, Buenos Aires, Argentina; ^9^Department of Pediatric Surgery, Necker-Enfants Malades Hospital, Université de Paris, Paris, France; ^10^Department of Pediatric Otolaryngology, Necker-Enfants Malades Hospital, Université de Paris, Paris, France; ^11^Department of Pediatric Oncology Surgery and Pediatric Oncology, Pediatric Oncology Institute—GRACC, Federal University of São Paulo, São Paulo, Brazil; ^12^Department of Head and Neck Surgery, Federal University of São Paulo, São Paulo, Brazil; ^13^Department of Pediatric Surgery, Alder Hey Children's Hospital NHS Foundation Trust, Liverpool, United Kingdom; ^14^Department of Pathology, Alder Hey Children's Hospital NHS Foundation Trust, Liverpool, United Kingdom; ^15^Department of Pediatric Surgery, Faculty of Health and Life Sciences, Alder Hey Children's Hospital NHS Foundation Trust, University of Liverpool, Liverpool, United Kingdom

**Keywords:** thyroid, cancer, children, surgery, carcinoma

## Abstract

**Background:**

Thyroid gland malignancies are rare in pediatric patients (0.7% of tumors); only 1.8% are observed in patients aged <20 years, with a higher prevalence recorded in women and adolescents. Risk factors include genetic syndromes, MEN disorders, autoimmune diseases, and exposure to ionizing radiation. Radiotherapy is also associated with an increased risk of secondary thyroid cancer. This study describes the clinical features and surgical outcomes of primary and secondary thyroid tumors in pediatric patients.

**Methods:**

Institutional data were collected from eight international surgical oncology centers for pediatric patients with thyroid cancer between 2000 and 2020. Statistical analyses were performed using the GraphPad Prism software.

**Results:**

Among 255 total cases of thyroid cancer, only 13 (5.1%) were secondary tumors. Primary thyroid malignancies were more likely to be multifocal in origin (odds ratio [OR] 1.993, 95% confidence interval [CI].7466–5.132, *p* = 0.2323), have bilateral glandular location (OR 2.847, 95% CI.6835–12.68, *p* = 0.2648), and be metastatic at first diagnosis (OR 1.259, 95% CI.3267–5.696, *p* > 0.999). Secondary tumors showed a higher incidence of disease relapse (OR 1.556, 95% CI.4579-5.57, *p* = 0.4525) and surgical complications (OR 2.042, 95% CI 0.7917–5.221, *p* = 0.1614), including hypoparathyroidism and recurrent laryngeal nerve injury. The overall survival (OS) was 99% at 1 year and 97% after 10 years. No EFS differences were evident between the primary and secondary tumors (chi-square 0.7307, *p* = 0.39026).

**Conclusions:**

This multicenter study demonstrated excellent survival in pediatric thyroid malignancies. Secondary tumors exhibited greater disease relapse (15.8 vs. 10.5%) and a higher incidence of surgical complications (36.8 vs. 22.2%).

## Introduction

Thyroid tumors are rare malignancies in children, accounting for only 0.7% of all pediatric cancers (1). In the United States, only 1.8% of thyroid tumors are diagnosed in patients younger than 20 years of age, with a notable prevalence in women, adolescents (2), and Caucasians (3). Many risk factors have been linked to the development of thyroid cancer, including Hashimoto's thyroiditis (4), genetic disorders [such as multiple endocrine neoplasia type 2, Carney's syndrome (5), Werner's syndrome (6), and DICER1 syndrome (7)], and a history of exposure to ionizing radiation (8).

The correlation between radiation exposure and an increased risk of childhood thyroid cancer was demonstrated in patients after the Chernobyl nuclear accident in 1986 (8). However, it should also be noted that patients with cancer treated with therapeutic radiation (external beam for neck cancers and total-body radiation for leukemias and lymphomas) are reportedly “at risk” for secondary thyroid malignancies (almost 2% of cases) (9–12). Indeed, patients surviving Hodgkin's lymphoma are 18.3–22 times more likely to develop papillary thyroid carcinoma than the healthy general population (13).

The risk is much higher in younger patients (<5–10 years of age) (9, 14) and for dosimetry between 20 and 29 Gy (15). Higher doses of radiotherapy seem to be associated with a lower risk of neoplastic transformation of the thyroid gland, possibly because radiation intensity may induce a direct necrotic effect on thyroid cell viability (14). No differences in the incidence rates were reported according to sex (12, 16).

The latent interval between the first individual radiation exposure and thyroid cancer development is variable (0.5–38 years) (17) but is usually reported to be ~20 years (16). The most frequent neoplasms recorded were papillary (69–87%) and follicular (5–23%) carcinomas (18).

According to the contemporary literature, secondary thyroid tumors are more prone to be metastatic and multifocal at diagnosis (11, 18, 19), but, controversially, are not believed to be associated with a worse prognosis (18–20); there is currently no international consensus on individual phenotypic characteristics of secondary lesions (16, 21).

Although pediatric thyroid surgery is increasingly recognized as “low-volume activity,” it is nonetheless crucial for pediatric surgeons to be aware of thyroid neoplasia, as the incidence of these lesions increases in communities that may be co-associated with high rates of complications, notably hypoparathyroidism and recurrent laryngeal nerve injury (9, 22).

To the best of our knowledge, few studies have sought to accurately determine whether worse surgical outcomes exist for thyroid tumors in pediatric patients with a prior history of treatment-related radiation exposure. Therefore, this study examines the clinical features and surgical outcomes of primary and secondary thyroid tumors in pediatric patients related to the background of current world literature on this important topic.

## Materials and methods

The authors retrospectively collected data on pediatric patients (<18 years of age) treated for thyroid cancer at eight participating international collaborative surgical oncology centers between 2000 and 2020:

- Bambino Gesù Children's Hospital in Rome—Italy
- University of Pisa—Italy
- University and Hospital Trust of Verona—Italy
- National Cancer Institute in Bogotá—Columbia
- Hospital Italiano de Buenos Aires—Argentina
- Necker-Enfants Malades Hospital in Paris—France
- Pediatric Oncology Institute—GRAACC—Federal University of São Paulo—Brazil
- Alder Hey Children's Hospital Liverpool—UK.

The patients were categorized into two distinct cohort study groups (primary and secondary tumors). Demographics, clinical outcomes, surgical outcomes, and survival data were analyzed.

Statistical analysis was performed using Fisher's exact test and the Gehan-Breslow-Wilcoxon test. Event-free survival (EFS) and overall survival (OS) were calculated using GraphPad Prism Version 8.

The study involving human participants was reviewed and approved by Institutional Ethical Committee. Written informed consent from the patients or legal guardians was not required to participate in this study in accordance with national legislation and institutional requirements.

## Results

A total of 255 patients were identified, 13 (5.1 %) of whom had previous malignancies (Table 1). The median latent interval between primary cancer treatment and subsequent development of a thyroid tumor was seven years (range 0.1–16 years).

**Table 1:** Characteristics of patients with secondary thyroid tumors.

**Case**	**Previous**	**Previous**	**Latency**	**Metastasis**	**Surgical**	**Histology**	**Surgical**	**Relapse**
	**malignancy**	**radiotherapy**	**(years)**	**at diagnosis**	**procedure**		**complications**	
1	Hodgkin Lymphoma	Yes	8	No	Hemithyroidectomy (then completed)	Papillary	No	No
2	T leukemia	Yes	11	No	Hemithyroidectomy (then completed)	Papillary	Yes (Hypoparathyroidism)	Yes (Bone)
3	Hodgkin Lymphoma	Yes	7	No	Hemithyroidectomy (then completed)	Papillary	No	No
4	Acute Lymphoblastic Leukemia	No	16	No	Total thyroidectomy	Adenoma	No	No
5	Acute Lymphoblastic Leukemia	Yes	5	No	Total thyroidectomy	Papillary	Yes (Hypoparathyroidism and lesion of laryngeal nerve)	No
6	Acute Lymphoblastic Leukemia	Yes	9	No	Hemithyroidectomy	Adenoma	No	No
7	Acute Lymphoblastic Leukemia	No	4	No	Total thyroidectomy	Papillary	Yes (Hypoparathyroidism)	No
8	Neuroblastoma	Yes	13	No	Hemithyroidectomy	Adenoma	No	No
9	Ovarian Sertoli Tumor	No	0.1	Yes (pulmonary)	Total thyroidectomy	Papillary	Yes (Hypoparathyroidism)	Yes (Cervical lymph nodes)
10	Ovarian mature teratoma	No	0.1	No	Total thyroidectomy	Papillary	No	No
11	Wilms tumor	No	7	No	Total thyroidectomy	Papillary	No	No
12	Thyroid Adenoma	No	6	Yes (vertebral)	Total thyroidectomy	Follicular	Yes (Hypoparathyroidism)	Yes (Bone)
13	Rhabdomyosarcoma	Yes	5	No	Total thyroidectomy	Papillary	No	No

Demographic characteristics are shown in Table 2. Seventy-eight (30.6%) patients were male; the median age at diagnosis was 14 years (range 1–18.4 years; IQR: 5 years) for those with primary thyroid tumors and 14 years (range 6–17.8 years; IQR: 4.1 years) for patients with secondary thyroid malignancies. Fifty-seven patients (22.4%) presented with associated pathologies or identifiable syndromes or disorders (Table 3). Most neoplasms (166 cases; 65.1%) were clinically detected as a “painless” thyroid nodule or mass. In 69 cases (27.1%), the lesions were impalpable and were discovered incidentally in imaging studies performed for other purposes. Only 25 patients (9.8%) presented with Thyroid-stimulating hormone (TSH) alteration. The median tumor size was 2 cm (range 0.1–12 cm).

**Table 2:** Demographics of patients.

	**Primary tumors**	**Secondary tumors**	**Total**
Male	71 (29.3%)	7 (53.8%)	78 (30.6%)
Race			
Caucasian	151 (62.4%)	10 (76.9%)	161 (63.1%)
Hispanic	60 (24.8%)	2 (15.4%)	62 (24.3%)
Black	7 (2.9%)	0	7 (2.7%)
Unknown	24 (9%)	1 (7.7%)	25 (9.8%)
Age at diagnosis (years)	14 (1–18.4)	14 (6–17.8)	14 (1–18.4)

**Table 3:** List of associated pathologies and syndromes.

**Associated pathology/syndrome**	**Number of patients**
Hashimoto's thyroiditis	14
MEN Syndrome type 2	13
Multinodular goiter	7
Basedow's disease	3
Epilepsy	2
Rheumatoid arthritis	1
Congenital Hyperinsulinism	1
Hyperthyroidism	1
Endometriosis	1
Agenesis of left lobe thyroid	1
Congenital heart disease	1
Asthma	1
Pilonidal sinus	1
Hepatic adenoma	1
Kabuki Syndrome	1
Tuberous sclerosis	1
Trisomy 21	1
Type 1 Diabetes	1
Autoimmune hepatitis	1
Thalassemia major	1
Lymphatic abdominal wall malformation	1
Cowden syndrome	1
Turner syndrome	1

Concerning the tumor characteristics at the index diagnosis, 56 primary tumors (23.1%) involved both thyroid lobes. Primary tumors were more prone to multifocality at the first diagnosis (98 patients, 40.5%) than secondary lesions (2 cases, 18.2%). Fisher's exact test showed no statistically significant differences in terms of bilaterality (OR 0.3513, 95% CI 0.07886–1.463; *p* = 0.2648) and multifocality (OR 1.993, 95% CI 0.7466–5.132; *p* = 0.2323) when comparing primary and secondary tumors.

A total of 36 patients (14.1%) showed metastasis at diagnosis: 32 (94.1%) had distant metastasis in the lung, one case (2.8%) in the bone vertebra, and one case (2.8%) had lung and bone dissemination. Primary thyroid malignancies were more likely to be metastatic at presentation (OR 1.259, 95% CI, 0.3276–5.696; *p* > 0.999 n.s.).

A total of 214 patients (83.9%) underwent fine-needle aspiration biopsy (FNAB) for tissue diagnosis. Cytopathology was reported according to the Bethesda System as follows: 3.4% Tyr1, 5.2% Tyr2, 18.3% Tyr3, 22.9% Tyr4, 23.4% Tyr5, and 26.9% Tyr6.

Almost all the patients (*N* = 219, 85.9%) underwent total thyroidectomy. In 36 cases (14.1%), a hemithyroidectomy was initially performed, but based on tumor pathology, 18 of these patients (50%) underwent a subsequent completion thyroidectomy. In a single patient, thyroid cancer was incidentally detected within the resected pathology specimen of a thyroglossal duct cyst after the Sistrunk operation. Due to pathology findings of aggressive papillary carcinoma, the patient underwent a complete thyroidectomy.

In 153 (60%) patients, lymphadenectomy was performed during the initial surgery, based on the perioperative or US imaging findings. Of these, 131 (85.6%) showed positive lymph node pathology.

A total of 218 (85.5%) patients had papillary carcinoma, 13 (5.1%) had thyroid adenomas, 11 (4.3%) had follicular carcinoma, 12 (4.7%) had medullary carcinoma, and a single patient (0.3%) had thyroid lymphoma.

Secondary thyroid tumors showed a higher rate of surgical complications (OR 2.042, 95% CI.7917–5.221, *p* = 0.1614), including hypoparathyroidism (OR 1.838, 95% CI.6763–4.822, *p* = 0.2452) and recurrent laryngeal nerve injury (OR 1.218, 95% CI.2654–4.902, *p* = 0.6816). Four patients with primary malignancies required additional surgery due to postoperative complications. Two of them had postoperative bleeding and two suffered respiratory distress that was managed with a temporary tracheotomy.

After a median follow-up of 3 years (range 0–18 years), 27 patients (10.6%) showed evidence of disease relapse:21 patients had recurrence at the lymph node sites, three had distant lung metastases, two had bone involvement, and one had metastases in both the lungs and lymph nodes. Recurrence sites in lymph nodes were mostly ipsilateral (57.1%); only in three cases (14.3%) the relapse was identified in the contralateral lymph nodes and in four patients (19%) on both sides. Secondary thyroid tumors showed a non-significant trend towards a higher incidence of disease relapse (OR 1.556, 95% CI.4579–5.57, *p* = 0.4525).

A lethal outcome was observed in three patients (1%) with primary thyroid malignancies. Two died due to disease progression and one from cerebral ischemia unrelated to the thyroid disease. The latter patient had hypoplastic left heart syndrome and underwent the First Stage Norwood Procedure + Bilateral Glenn Shunt in 1996 and Extracardiac Fontan Procedure in 1997. The ascending aorta graft showed aneurysmatic degeneration in 2016, requiring its substitution in 2017. During this procedure, the patient suffered from massive intraoperative cerebral ischemia and brain death was declared.

**Figure 1:**
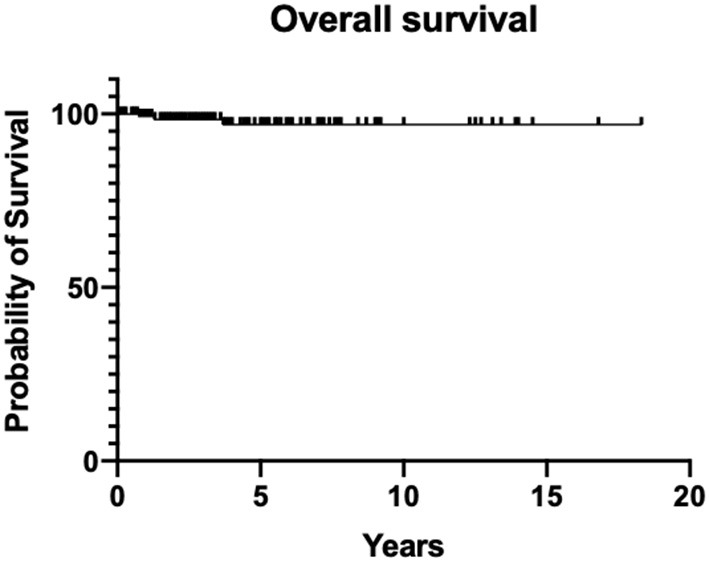
Overall survival (OS) of the study population.

**Figure 2:**
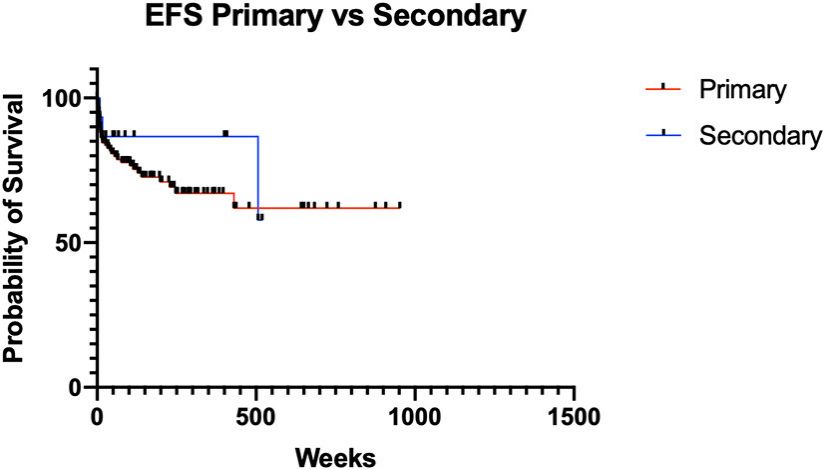
Event free survival (EFS) for primary and secondary thyroid neoplasms.

The overall survival (OS) in the study population was generally excellent, with 99% at 1 year and 97% after 5 and 10 years (Kaplan Meier, Figure 1). Event-free survival (EFS) differences were not observed between primary and secondary thyroid neoplasms—(*X*^2^ Chi-square 0.7307, *p* = 0.39026) (Figure 2).

## Discussion

Thyroid cancer is a rare malignancy in children and accounts for only 0.7% of all pediatric tumors (1). Many risk factors that predispose individuals to the development of malignant lesions of the thyroid gland have been identified, notably Hashimoto's thyroiditis (4), genetic disorders, multiple endocrine neoplasia syndrome type 2, and ionizing radiation exposure (8).

The thyroid gland is particularly vulnerable to the carcinogenic effect of exposure to ionizing radiation (14) and, therefore, pediatric patients having prior radiotherapy are of critical concern to healthcare professionals. In a recent report from the Childhood Cancer Survivor Study, those with Hodgkin's lymphoma were 18.3–22 times more likely to acquire papillary thyroid cancer than the general healthy population (13, 16). The risk of developing radiation-induced thyroid cancer is dependent on numerous factors, including the age of the child at the time of first treatment, radiation dosimetry, and time elapsed since radiation treatments.

The minimum latency period reported in the literature was 5 years, with a peak at ~15–30 years. Thereafter, the risk appears to decline, although it is still apparent even after 40 years (11).

The individual risk of secondary thyroid tumors in patients exposed to ionizing radiation (for medical purposes or from a nuclear accident) increases significantly after a mean dose of 20–29 cGy (15), with a linear relationship between the dosage and the risk of carcinoma. At doses higher than 1,500 cGy, the relative risk oddly decreases, theoretically assumed to be caused by radiation-induced thyroid gland cellular necrosis (11).

Younger age in radiotherapy has also been described as a major risk factor. It is maximal when exposure occurs under the age of 5 years, and this risk then decreases in older age groups (11).

Although thyroid cancer from radiation exposure has been well-described, there is no unanimity regarding the specific disease characteristics resulting from ionization in humans. According to some experts, secondary thyroid cancer lesions are more prone to be multifocal (11) and metastatic (19, 23) at diagnosis; however, other authors contradict such claims (16, 21). In this international collaborative study, primary tumors were observed in (a) bilateral lobar sites in the thyroid gland (OR 2.847, *p* = 0.2648), (b) multifocal in profile (OR 1.993, *p* = 0.2323), and (c) metastatic sites (OR 1.259, *p* > 0.999) at first diagnosis. Dependent on the ultrasound imaging features of the thyroid nodule (i.e., hypoechogenicity, irregular margins, increased blood flow, and microcalcifications) and patient characteristics, a decision pathway is then deployed by clinicians as to whether fine-needle aspiration biopsy (FNAB) of the nodule is warranted (1). In the study cohort population, we analyzed 214 patients (83.9%) who underwent FNAB with a preponderance of suspicious/malignant lesions (Bethesda staging: 22.9% Tyr4, 23.4% Tyr5, and 26.9% Tyr6). In all the remaining cases gathered from the collaborating centers, “up front” surgical excision of the lesion was directly performed. According to the 2015 American Thyroid Association (ATA) guidelines (4), thyroid lobectomy may be permissibly scheduled in patients with compressive physiological symptoms, in those with apparently benign solid thyroid nodules of >4 cm in size with significant growth, and/or in the presence of other clinical concerns for malignancy. Regarding surgical approaches, the current recommendations from the ATA for pediatric thyroid nodules are total thyroidectomy (4) to reduce the risk of persistent or recurrent thyroid disease. However, some authors have suggested hemithyroidectomy in intermediate- and low-risk populations (24, 25), stratified according to the GAMES classification system (26). This system was published in 1994 after a detailed analysis of 1,038 patients with DTC attending the Memorial Sloan Kettering Cancer Center, USA, which stratified patients into low-, intermediate-, and high-risk categories based on grade, age, distant metastases, extrathyroidal extent, and size. In this international collaborative study, 85.9% of patients underwent total thyroidectomy, with 14.1% of patients undergoing hemithyroidectomy (with total thyroidectomy occurring later in 50% of those cases). A single patient in this study had a papillary carcinoma detected in a thyroglossal duct cyst after a Sistrunk's operation; this case then required “curative” definitive total thyroidectomy (27).

According to the international care guidelines, prophylactic central and lateral neck dissection is not generally recommended (4). It has been stated that this should only be undertaken in patients with malignant cytology (Bethesda stages 4, 5, or 6), or where there is frank clinical evidence of extrathyroidal nodal disease invasion or locoregional metastasis from preoperative staging investigations, or results from intraoperative findings. In this study, 153 pediatric patients (60%) underwent lymphadenectomy, with 85.6% showing lymph node metastasis.

Our results on tumor biology are in accordance with, so far, published studies (1): the most common tumors encountered were papillary cancers (85.5%), thyroid adenomas (5.1%), follicular lesions (4.3%), and medullary tumors (4.7%). We also report a rare case of thyroid lymphoma.

Pediatric thyroid surgery represents “low-volume activity” and should only be performed by skilled surgical teams with shared considerations for active collaboration with an adult endocrine surgeon (4, 22). Risks of thyroid surgery are well-known, including hemorrhage, hypocalcemia resulting from parathyroid injury or inadvertent removal, and recurrent laryngeal nerve injury. The risk of developing transient hypocalcemia after total thyroidectomy is estimated to be ~52%, and it is believed to be much higher in patients with an extensive bulky disease requiring more extensive surgery. Fortunately, it is rarely permanent (<2%) in “high-volume” adult surgical endocrine practice (28, 29). Reported rates of injury to the recurrent laryngeal nerve leading to significant palsy range between 2 and 6%. The wider adoption of intraoperative laryngeal nerve monitoring, however, may help offset these problems (30). In this study, surgical morbidity was reported in 64 cases (25.1%): 59 (24.4%) patients had primary tumors, and 5 (38.5%) patients had secondary tumors. There was a trend towards a higher incidence of surgical morbidity in the group with secondary thyroid tumors (OR 2.042, *p* = 0.1614).

In this study, hypoparathyroidism (OR 1.838, *p* = 0.2452) and recurrent laryngeal nerve injuries (OR 1.218, *p* = 0.6816) seemed to be more frequent in patients with previous malignancies. These findings may be explained, in part, by the frequent presence of dense adhesions of the thyroid gland to nearby anatomical structures in patients who had previous neck radiotherapy, thus, presenting difficult challenges during surgery.

In this study, only patients with primary thyroid neoplasms required a “second look”, or additional procedures for surgical complications, notably securing hemostasis (*n* = 2), and temporary tracheostomies (*n* = 2) for acute respiratory distress disorders.

We herein confirm, as reported by Shaha (19), that differentiated thyroid cancer occurring in patients with a history of radiation treatment showed similar survival outcome metrics to those with primary thyroid tumors. Although secondary thyroid tumors showed a higher risk of relapse (OR 1.556, *p* = 0.4525), there were no statistically significant differences in EFS reported (chi-square 0.7307, *p* = 0.39026).

As with many retrospective cohort studies, we acknowledge that this study had several limitations. The participating study group centers, for example, are likely to be subject to variation in clinical practice outcomes linked to hospital volume activity and the skills of each surgical team with rare diseases. Surgical techniques may also differ according to the specialty field of the surgeon and teams, notably (a) pediatric surgeons, (b) pediatric surgeons with adult endocrine surgeons, or (c) otorhinolaryngology (ENT) specialists.

In summary, no statistical differences in outcome metrics following thyroid surgery were observed in this study when comparing patients diagnosed with primary and secondary tumors.

This multicenter collaborative report further demonstrated excellent survival for pediatric thyroid malignancy in patients with primary and secondary neoplasms.

## Data availability statement

The raw data supporting the conclusions of this article will be made available by the authors, without undue reservation.

## Author contributions

CM, AC, and AI conceived the study and wrote the first draft of the manuscript. PL worked on the multiple drafts and re-edits of the manuscript. CM had final responsibility for the decision to submit for publication. All the authors contributed to the study design, analysis, data collection, and interpretation of the results, reviewed the manuscript, and agreed with the decision to submit it for publication.

## Conflict of interest

The authors declare that the research was conducted in the absence of any commercial or financial relationships that could be construed as a potential conflict of interest.

## Publisher's note

All claims expressed in this article are solely those of the authors and do not necessarily represent those of their affiliated organizations, or those of the publisher, the editors and the reviewers. Any product that may be evaluated in this article, or claim that may be made by its manufacturer, is not guaranteed or endorsed by the publisher.
